# Plasma Biomarkers for Cerebral Amyloid Angiopathy and Implications for Amyloid-Related Imaging Abnormalities: A Comprehensive Review

**DOI:** 10.3390/jcm14041070

**Published:** 2025-02-07

**Authors:** Mo-Kyung Sin, Jeffrey L. Dage, Kwangsik Nho, N. Maritza Dowling, Nicholas T. Seyfried, David A. Bennett, Allan I. Levey, Ali Ahmed

**Affiliations:** 1 College of Nursing, Seattle University, Seattle, WA 98122, USA; 2 School of Medicine, Indiana University, Indianapolis, IN 46202, USA; jdage@iu.edu (J.L.D.); knho@iu.edu (K.N.); 3 School of Nursing, George Washington University, Washington, DC 20052, USA; nmdowling@gwu.edu; 4 Department of Biochemistry, School of Medicine, Emory University, Atlanta, GA 30329, USA; nseyfri@emory.edu; 5 School of Medicine, Rush University, Chicago, IL 60612, USA; david_a_bennett@rush.edu; 6 School of Medicine, Emory University, Atlanta, GA 30322, USA; alevey@emory.edu; 7 Department of Medicine, Veterans Affairs Medical Center, Washington, DC 20422, USA; ali.ahmed@va.gov; 8 Department of Medicine, George Washington University, Washington, DC 20037, USA; 9 Department of Medicine, Georgetown University, Washington, DC 20057, USA

**Keywords:** Alzheimer’s disease, ARIA, anti-amyloid therapy, biomarkers, cerebral amyloid angiopathy, plasma

## Abstract

Anti-amyloid therapies (AATs) are increasingly being recognized as promising treatment options for Alzheimer’s disease (AD). Amyloid-related imaging abnormalities (ARIAs), small areas of edema and microbleeds in the brain presenting as abnormal signals in MRIs of the brain for patients with AD, are the most common side effects of AATs. While most ARIAs are asymptomatic, they can be associated with symptoms like nausea, headache, confusion, and gait instability and, less commonly, with more serious complications such as seizures and death. Cerebral amyloid angiopathy (CAA) has been found to be a major risk for ARIA development. The identification of sensitive and reliable non-invasive biomarkers for CAA has been an area of AD research over the years, but with the approval of AATs, this area has taken on a new urgency. This comprehensive review highlights several potential biomarkers, such as Aβ40, Aβ40/42, phosphorylated-tau217, neurofilament light chain, glial fibrillary acidic protein, secreted phosphoprotein 1, placental growth factor, triggering receptor expressed on myeloid cells 2, cluster of differentiation 163, proteomics, and microRNA. Identifying and staging CAA even before its consequences can be detected via neuroimaging are critical to allow clinicians to judiciously select appropriate candidates for AATs, stratify monitoring, properly manage therapeutic regimens for those experiencing symptomatic ARIAs, and optimize the treatment to achieve the best outcomes. Future studies can test potential plasma biomarkers in human beings and evaluate predictive values of individual markers for CAA severity.

## 1. Introduction

In the United States (U.S.), Alzheimer’s disease (AD) is the 5th leading cause of death [1] and the most common cause of dementia among older adults [2]. AD affected approximately 6.9 million Americans aged 65 and older in 2024 [1]. Considering the growing number of older adults, the prevalence and incidence of AD are expected to increase, with estimations indicating that nearly 13 million people will be affected by 2050 [1]. Anti-amyloid therapies (AATs) are approved by the U.S. Food and Drug Administration and have become promising treatment options for AD [3,4,5]. However, like other medications, AATs have side effects. Amyloid-related imaging abnormalities (ARIAs), i.e., abnormal signals seen in MRIs of the brain for patients with AD, are the most common side effect of AATs. Cerebral amyloid angiopathy (CAA) has been found to be a major risk for ARIA occurrence. The identification of sensitive and reliable non-invasive biomarkers for CAA has become a current trend of AD research following the approval of AATs. There have been great advances in the development of blood-based biomarkers that can help us to understand AD. Blood-based testing is a rapid, minimally invasive, and inexpensive method with high accuracy in identifying AD [6,7,8]. However, very few studies aiming to identify blood-based biomarkers associated with the development or severity of CAA have been conducted. In this review, we aim to explain why this is an urgent area of research, highlight potential candidate blood-based biomarkers (amyloid β, tau, glial fibrillary acidic protein, neurofilament light chain, secreted phosphoprotein 1, placental growth factor, triggering receptor expressed on myeloid cells 2, cluster of differentiation 163, proteomics, and microRNA), and outline a discovery strategy for identifying novel blood-based biomarkers for CAA.

## 2. AD, AATs, and ARIAs

The first sign of AD is the accumulation of amyloid β plaques in the brain. AD typically advances from preclinical AD to mild cognitive impairment (MCI) and potentially dementia due to AD (Table 1) [1,9].

Each individual spends a different amount of time in each stage. For example, influenced by age, genetics, biological sex, clinical setting, and baseline CSF t-tau levels, the total disease duration varies between 12 and 25 years, while the duration of the preclinical stage varies between 2 and 15 years, the length of the MCI stage varies between 3 to 7 years, the duration of the mild AD dementia stage varies between 2 and 6 years, and the length of the moderate AD dementia stage varies between 1 and 7 years [9]. Older people, males, ApoE Ɛ4 carriers, and those with increased CSF t-tau levels experience faster cognitive decline with a shorter duration [9]. Identifying ways to slow down cognitive decline and prolong the duration within a milder disease stage is desperately needed to increase the quality of health of older adults.

Approved and experimental monoclonal antibodies designed to target Aβ plaques and their precursor molecules have proven to be promising therapeutic tools with beneficial effects on individuals with mild cognitive impairment and mild dementia due to AD in randomized controlled trials [3,4,5]. The FDA-approved anti-amyloid drugs include lecanemab, aducanumab, and donanemab. ARIAs are the most common side effect of AATs, and they are classified into two subtypes: ARIA-E (edema/effusion) and ARIA-H (microhemorrhages, macrohemorrhages and/or superficial siderosis) [4,10]. The common clinical symptoms of ARIAs include changes in mental status, confusion, headache, visual disturbances, vomiting, nausea, gait disturbances, tremor, and even death [4,5,10,11]. Within the first 3 months for 10 mg/kg monthly and biweekly dosing regimens in a phase 2 lecanemab trial, the incidence of ARIA-E was <10% and that for symptomatic ARIA was <3% [3,10]. In a phase 3 lecanemab trial, the incidence of ARIA-E was 12.6% (vs. 1.7% with placebo) and that for ARIA-H was 17.3% (vs. 9.05% with placebo) [5]. As AATs are becoming promising treatment avenues, improved knowledge of the pathophysiology of ARIA is necessary for the early detection of those who are at greatest risk.

## 3. Risk Factors for ARIA Development

Several risk factors for ARIA, such as CAA; the presence of one or more microhemorrhages/superficial siderosis and white matter disease at baseline; a higher baseline amyloid load; hypertension [12]; the initial treatment period (e.g., most instances of ARIA-E occur within the first 3 months of treatment); the type of antibody used [13,14,15,16,17,18,19,20]; higher dosages [11]; an ApoE Ɛ4 genotype, with ApoE Ɛ4 homozygotes posing the greatest risk [15,21,22,23,24]; and anti-thrombin use [12], have been identified. Because CAA is highly prevalent in AD (up to 93.6%) [25] and both conditions have the same underlying mechanism, namely, impaired Aβ clearance [24], CAA is a key risk factor for ARIA development.

The high prevalence of CAA in AD places many patients at risk for ARIA-associated morbidity. ARIAs can be serious (e.g., leading to hospitalization, disability, etc.) and life-threatening for approximately 1% of patients [11,26,27]. The morbidity and cost of care can be substantial. This highlights the importance of detecting and staging CAA in its “preclinical” stage, even before its consequences can be detected on neuroimaging. However, the ability to identify people with preclinical CAA is severely limited due to a lack of a reliable, specific, and sensitive screening tool.

Histopathological confirmation via brain biopsy or autopsy, which is not clinically feasible, is the gold standard for CAA diagnosis [28]. Among in vivo tools, CT and MRI are most reliable in identifying CAA according to the modified Boston criteria [29], but these approaches detect only the secondary consequences of CAA [30]. CAA is often asymptomatic. Its presence may be detected in vivo through surrogate telltale signs in MRIs, such as edema or hemorrhaging, which can present as micro- or macro-hemorrhages or superficial siderosis [3,5,10,31]. The modified Boston criteria offer one schema for identifying CAA [32] but only in the presence of bleeding. In addition, the imaging markers lack specificity because they may be partially induced by arteriosclerotic small-vessel disease [33]. Amyloid PET imaging has limited diagnostic accuracy for CAA because it is unable to differentiate between vascular and parenchymal amyloid β (Aβ) [34].

## 4. Diagnosis of CAA

**Figure 1:**
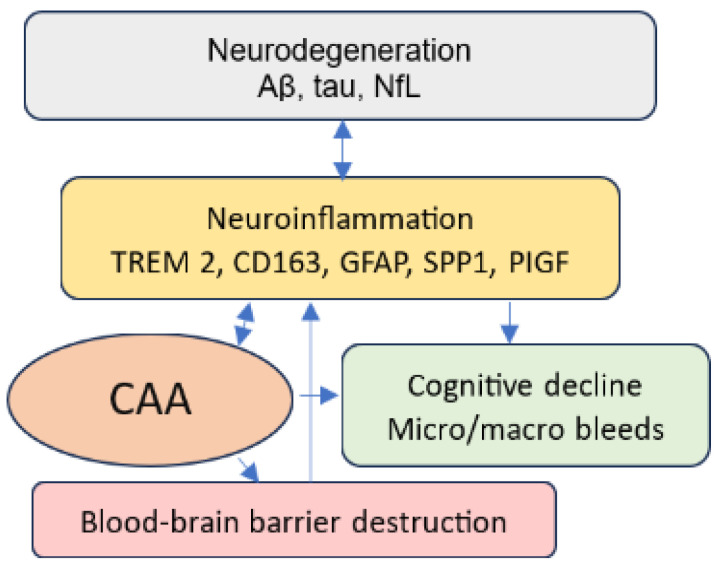
Neurodegeneration, neuroinflammation, and CAA.

The identification of sensitive and reliable non-invasive biomarkers for CAA has been an area of AD research for some time, but with the approval of AAT, research in this area has taken on a new urgency. The Foundation of the NIH Biomarkers Consortium reported that plasma Aβ is highly correlated with amyloid PET or CSF amyloid β (Aβ) levels [35]. However, plasma tests have mainly been used to detect AD, not CAA. A recent study conducted on animals found reduced Aβ40 levels in CSF and plasma in early-stage CAA prior to the onset of cerebral microbleeds as assessed via MRI and histological staining; then, Aβ40 levels abruptly dropped at the early onset of CAA and continued to decrease as CAA progressed [36]. Considering the advantages of using plasma tests and the promising study findings, further studies measuring Aβ peptide levels in humans for CAA diagnosis are needed to advance AD management. However, there is a need to explore the relationship between CAA and parenchymal amyloid plaque, and this endeavor requires the use of a multi-biomarker panel. Thus, in this review, we propose to investigate several potential biomarkers for CAA identification, such as Aβ40, Aβ40/42, phosphorylated-tau217 (p-tau217), neurofilament light chain (NfL), glial fibrillary acidic protein (GFAP), secreted phosphoprotein 1 (SPP1), placental growth factor (PIGF), triggering receptor expressed on myeloid cells 2 (TREM2), cluster of differentiation (CD)163, proteomics, and microRNA. Figure 1 depicts the pathways of the potential biomarkers to CAA via neurodegeneration, neuroinflammation, and pathological angiogenesis.

Neurodegeneration, neuroinflammation, and pathological angiogenesis are direct consequences of excess Aβ in the brain. They are frequently seen in AD brains and mutually reinforcing [37,38]. The presence of Aβ and tau in neurodegenerative disease triggers and exacerbates neuroinflammation by activating glial cells, such as microglia and astrocytes, and immune cells, such as macrophages. Both microglia and perivascular macrophages play important roles in Aβ clearance in parenchyma and CAA [39]. Activated microglia, macrophages, and neuroinflammation can also exacerbate Aβ deposition, resulting in a vicious cycle. The presence of Aβ in the blood vessels is related to neuroinflammation and vessel inflammation, contributing to blood–brain barrier destruction, which further leads to neuroinflammation and cognitive impairment.

Aβ, made up of 39–43-residue amyloidogenic peptides (including Aβ1-40 and Aβ1-42), are produced from amyloid precursor protein [40,41]. Soluble Aβ is produced throughout one’s life, but some of it is pathologically converted into insoluble Aβ, which is the major component of the neuritic plaques in the brain parenchyma and vessel Aβ [42]. Neuritic plaques, the result of Aβ accumulation in the brain parenchyma, are a pathological hallmark of AD [40,43]. In contrast, CAA is the accumulation of Aβ in cerebral vessels, primarily in the medium-sized arteries and arterioles in the leptomeningeal and cortical regions [42,44,45,46,47,48]. Advanced CAA can render vessels fragile and prone to rupturing, resulting in intracerebral hemorrhages in lobar regions, cortical superficial siderosis, and white matter hyperintensities [36,49,50,51,52,53]. Cerebral microhemorrhages, cortical superficial siderosis, and a history of intracranial hemorrhage are well-established surrogate markers of CAA severity [54]. While Aβ42 is thought to be the foundational seed for both parenchymal plaques and CAA formation, higher cerebral Aβ40 levels and Aβ40/42 ratios are more likely related to CAA formation, whereas higher cerebral Aβ42 and Aβ42/40 levels are more likely related to parenchymal plaques [44,45,46].

Aβ40 and Aβ42 reflect amyloid precursor protein metabolism and p-tau for tangle pathology [28]. Since CAA is the result of impaired clearance of Aβ from interstitial cerebral fluid, CSF contains biomarkers reflecting this process [34]. Several studies have tested CSF biomarkers such as Aβ40 and Aβ42 to identify early-stage CAA [33,34,55,56,57]. Table 2 shows CSF Aβ40, Aβ42, and Aβ40/42 levels with respect to individuals with CAA and AD [30]. All these studies agreed that people with CAA had lower CSF Aß40 and Aß42 levels than both the control and AD groups, but they were inconclusive on the ability to distinguish CAA from AD [56]. None of these studies included the use of a multi-biomarker panel to aid in the differentiation of CAA from AD. P-tau refers to tau proteins that have undergone phosphorylation [58]. Plasma p-tau 217 is one of the most promising biomarkers for diagnosing AD [59,60]. For example, plasma p-tau 217 was found to offer high accuracy in identifying elevated Aβ levels (area under the curve [AUC]: 0.92–0.96; 95% CI, 0.89–0.99) and tau pathology (AUC, 0.93–0.97; 95% CI, 0.84–0.99) in 786 individuals from three single-center observational cohorts [59]. In another study, plasma p-tau 217 was found to be offer high accuracy (AUC, 0.80–0.91) in identifying Aβ pathology, determined either via Aβ positron emission tomography or CSF Aβ42/40 ratio [60]. A meta-analysis reported that the core CSF biomarkers (Aβ40, Aβ42, and p-tau) can serve as molecular biomarkers of CAA [28]. However, the relationship between plasma Aβ and p-tau in the context of CAA has not been extensively studied.

Glial fibrillary acidic protein (GFAP), an astrocytic marker, is a Food and Drug Administration-cleared marker used to treat mild traumatic brain injuries/concussions [61]. The GFAP blood test helps clinicians determine the need for computerized tomography for patients with a suspected head injury and helps prevent unnecessary neuroimaging [61]. GFAP has recently been reported to be a potential biomarker for AD [62,63,64,65]. Studies have found that patients with AD have significantly higher GFAP levels than healthy controls [62,63,64]. In addition, elevated GFAP levels have been associated with increased neuritic (*p* < 0.001) and diffuse plaque density or counts (*p* = 0.001) as well as with Braak stages and tangle counts (*p* < 0.05) [63]. Plasma GFAP was found to be able to detect AD more accurately than CSF GFAP [66,67]. In addition, both serum and CSF GFAP were found to be biomarkers for neuroaxonal damage and astrocytosis in CAA [68].

Neurofilament light chain (NfL) is a protein and considered to be a biomarker for several neurodegenerative diseases. NfL is mainly found in large-caliber myelinated axons, and it is essential for the transmission of electrical impulses [69]. A low amount of NfL is present in normal physiological conditions such as brain development, maturation, and aging, but a high amount of NfL is present in the cerebrospinal fluid and blood when axonal damage or neurodegeneration occurs [69]. NfL is a reliable biomarker of neuronal damage, dementia, stroke, and traumatic brain injuries, and it is released into the bloodstream and CSF when brain cells are damaged [70,71,72]. NfL can be a powerful tool for measuring and predicting AD progression but not diagnosing AD because brain cell damage can be caused by other factors [70,72]. Elevated plasma NfL levels were found to have an association with changes in global cognition, attention, and amyloid PET longitudinally [70]. Studying the severity of AD can give insights into how the severity of AD is related to or exacerbates CAA. A recent study found serum NfL to be a biomarker for advanced CAA [68], but more studies are needed to confirm this finding.

Secreted phosphoprotein 1 (SPP1), also known as osteopontin, plays a role in cell adhesion, migration, and inflammation [73]. Osteopontin functions as a “bridge builder” in the central nervous system (CNS). It is expressed in nearly all cells in the CNS and interacts with several different types of receptors and proteins [74]. The longitudinal Northern Manhattan Study (NOMAS) conducted on three groups of adults aged ≥ 40 with dementia and cerebral small vessel disease (CSVD), dementia with no CSVD, and no dementia and no CSVD reported that the highest levels of plasma oesteoponin were in the group with dementia and CSVD, supporting a significant association between osteoponin levels and dementia with evidence of CSVD [75]. In addition, the NOMAS reported there was a strong correlation between osteoponin and white matter hyperintensity volumes [75]. Studies have identified that elevated levels of SPP1 are associated with increased neuroinflammation and neurodegeneration; thus, SPP1 is upregulated in several neuro-disorders, including multiple sclerosis, AD, and age-related macular degeneration [75,76]. A study based on the ROSMAP reported SPP1 expression was associated with faster cognitive decline, greater odds of AD and CAA, and activated microglia and neuroinflammatory disorders [77].

Placental growth factor (PIGF) is part of the vascular endothelial growth factor family. It is released in the highest amounts in the placenta and decidual tissues, but it can also be detected in endothelial tissues and bone marrow erythroblasts as well as a variety of tissues such as lung and heart tissue, adipose tissues, and skeletal muscle [78,79]. PIGF is either present in low amounts or nonexistent in healthy tissues, but it is present in high concentrations in diseased conditions. PIGF potentially plays roles in wound healing, tumor growth, and collateral vessel formation when ischemia occurs [80]. PIGF plays a role in angiogenesis, especially in pathological angiogenesis and inflammation [78,79]. In addition, PIGF can help distinguish whether cognitive impairment is the result of AD or an issue involving other vascular issues [79]. A multisite observational cohort study reported that there is a significant association between PIGF and cognitive impairment associated with white matter injury and dementia [81]. Others have also found elevated PIGF levels in people with a higher burden of white matter injury and cerebral microbleeds associated with AD [82]. A pilot study indicated that Aβ regulates angiogenesis through PIGF expression [38]. Endothelial dysfunction caused by PIGF is a common condition in CAA, highlighting the complex relationship between CAA and PIGF in vascular pathology. GFAP, SPP1, and PIGF are emerging biomarkers for AD research, and understanding their relationships with CAA might provide insights into the mechanisms underlying vascular changes in CAA.

The central nervous system (CNS) contains numerous myeloid cells and defensive barriers such as the meninges, the perivascular space, and the choroid plexus [83]. The central myeloid cells include parenchymal microglia as well as non-parenchymal macrophages such as perivascular macrophages, meningeal macrophages, choroid plexus macrophages, and monocytes [83,84]. Microglia are glial cells and considered the tissue-resident macrophages of the CNS located in the parenchyma [77,85]. Microglia constitute 10–12% of CNS cells and perform three essential functions: sensing, housekeeping, and protection against injurious self- and foreign stimuli (host defense) [86]. Microglia sense and scan the surrounding area every few hours and rapidly polarize toward a focal injury. The housekeeping functions of microglia include engaging in synaptic remodeling, phagocytosing dead or dying cells by moving to the site of neuronal death, and maintaining myelin homeostasis. Microglia mediate the host’s defenses against pathogens by initiating neuroinflammatory responses (e.g., producing cytokines and chemokines, recruiting additional cells and inducing their activity to clear pathogens, and maintaining brain homeostats (a neuroprotective role)) [83,85]. These neuroinflammatory responses help CNS neural networks mature, leading to healthy CNS function [76]. Healthy brains are capable of engaging in these responses, but dysregulation of any of the three responses (e.g., via persistent neuroinflammation) can lead to imbalance and neurodegeneration. The emergence of disease-associated microglia (DAMs), along with unique gene expression signatures, is the consequence of neuroinflammation and neurodegeneration [76]. Microglial activation plays an important role in the pathophysiology of AD [87,88] and CAA-related inflammation [89,90], eventually leading to ARIA. Identifying the crucial factors influencing the shift from healthy microglia to DAMs might help in developing microglial-cell-based therapeutic approaches [91].

Microglia may have a protective effect on AD by limiting the spread of Aβ and tau pathologies, but they also have a detrimental effect when phagocytosing Aβ, as they further damage unaffected brain tissue [92]. Understanding the relationship between microglia and AD may hold the key to AD management.

Triggering receptor expressed on myeloid cells 2 (TREM2) is a major microglial activation marker and plays a crucial role in microglial survival, activation, phagocytosis, and the maintenance of brain homeostasis and inflammatory responses to injuries or neurodegeneration [93]. Several studies have found that TREM2 is a reliable biomarker of AD and a contributor to neuroinflammation [94,95,96,97]. In an AD mouse model study, the loss of TREM2 was associated with a remarkable increase in *Aβ* load in the brain but also a dramatic decrease in CAA, showing the different effects of TREM2 in parenchymal and vascular *Aβ* pathologies [97]. A study reported a positive association between the concentration of p-tau in the CSF and TREM2 levels [95]. Others have proposed a potential connection of TREM2 with ARIA [98]. TREM2 is expressed in microglia and macrophages, and thus CSF levels and blood levels may have unique characters. Measuring TREM2 as a blood-based biomarker may provide unique evidence for CAA, wherein the macrophages may have a more dominant role.

Cluster of differentiation 163 (CD163) is a macrophage activation marker and has anti-inflammatory properties [99]. CD163 was found to be correlated with small-vessel injury in early AD in a mouse model [99]. Both microglia and macrophage activation markers have been gaining attention, especially when the identification of CAA biomarkers is urgently needed for ARIA management [98]. An animal model study reported that increased perivascular macrophage (e.g., CD163) turnover promotes vascular Aβ clearance, suggesting that activating perivascular macrophage could be a potential therapeutic strategy for clearing vascular amyloids [100]. Macrophages are white blood cells that play a crucial role in immune surveillance (immune cells) and the phagocytosis of pathogens and cellular debris [39]. CNS-associated macrophages are found in the meninges (leptomeninges and dura) [85]. Activated by pathogen invasion, macrophages produce inflammatory cytokines and activate signaling pathways leading to tissue repair [101]. Both microglia and the phagocytic activity of perivascular macrophages have been found to play a significant role in Aβ clearance not only in parenchyma but also in cerebral vessels [39]. Many potential microglial and macrophage molecules have been identified and are currently being tested in preclinical models, and much more effort is needed to speed up the transition to the clinical setting. Table 3 below provides the results of our literature search for potential biomarkers.

Proteomics has remarkable potential with respect to advance clinical research and biomarker discovery. Studies have identified several proteins that can indicate amyloid pathology [104,105,106,107]. For example, a study identified specific groups of proteins differentially expressed in patients with CAA pathology and CAA rat models (e.g., rTg-DI) in the CSF [106]. They found that the cathepsin protein family (CTSB, CTSD, and CTSS) and its main inhibitor (CST3) in rats and synaptic proteins (e.g., VGF, NPTX1, and NRXN2) and several members of the granin family (SCG1, SCG2, SCG3, and SCG5) were differentially expressed in humans in comparison to the controls. Another proteomics study involving a laser dissection microscopy-assisted mass spectrometry analysis of post-mortem human brain tissue identified potentially highly selective markers of CAA (e.g., clusterin, apolipoprotein E (APOE), serum amyloid P-component, norrin, collagen and alpha-2(VI)) [105]. Considering the remarkable promise of proteomics in identifying amyloid pathology, further studies are needed to identify CAA in humans [91].

MicroRNAs are short (~22 nt-long) and constitute a class of non-coding RNAs [108] that play a role in gene expression [109]. MicroRNAs can be used for diagnostic or therapeutic purposes because of the ability to detect circulating microRNA (c-microRNA) [109]. C-microRNA is released when cells are injured, inflamed, necrotic, or apoptotic [109]. Several studies have reported associations between dysregulated microRNA expression and neurodegenerative diseases such as AD [110,111,112]. An RNA-sequencing study reported that microRNAs are most abundant in human plasma, making up over 42.34% of all raw reads and 76.20% of all mappable reads [113]. Since these microRNAs are transported throughout the body through “insulation”—unlike free-floating mRNAs, which are quickly degraded by RNases—they have significantly longer half-lives (which can be 5 days or longer in some cases), making these RNA transcripts great biomarker candidates for disease [109]. MicroRNAs function as modulators of both neuronal and immune processes (termed “neurimmiRs”) in the CNS [114]. MicroR-124, microR-132, and microR-146 are prototypes of microRNAs and play important roles in neurodegeneration, neurogenesis, and synaptic plasticity [115]. MicroRNAs have emerged as promising markers for understanding the underlying complex pathology of AD based on its involvement in Aβ metabolism, neuroinflammation, and synaptic function [108,116]. Because of its role in Aβ metabolism, microRNA may be a biomarker for CAA. More clinical studies are necessary.

## 5. Discussion

The advent of the use of AATs for the prevention and treatment of AD and the recognition that ARIAs might limit their usefulness have prompted a new line of research exploring how to better predict the AAT-related risk posed by ARIAs. Because CAA is a major risk factor posed by ARIAs, the identification of CAA and risk stratification based on CAA may help optimize AAT use. As discussed above, there are many potential biomarkers that can be used to identify and stage CAA even before its consequences can be detected via neuroimaging.

Identifying specific and sensitive diagnostic markers that can be used to identify patients with AD who are at greater risk of ARIA development prior to treatment with an AAT is of substantial importance. Even their use within AAT clinical trials is an urgent priority for maximizing patient safety and improving the risk-vs.-benefit trade-off of therapies. AATs cause serious (e.g., leading to hospitalization and disability) and life-threatening ARIAs in approximately 1% of patients [11,26,27]. The morbidity and cost of care for ARIAs can be substantial. In 2024, about 7 million Americans were living with AD [1]. If as few as one million received AAT, one can expect serious complications in 10,000 cases. The average adjusted cost of hospitalization per inpatient stay in 2019 was USD 14,101 [117]. The 65-and-older population is estimated to reach 82 million by 2050 [118], while cases of AD are expected to reach approximately 13 million by 2050 [1]. The healthcare costs for ARIA-related complications will escalate with the increasing number of older adults and the growing number of patients with AD starting AATs. In addition, there is no alternative to AATs with respect to treatment. Therefore, it is of upmost importance to study which patients will be prone to these side effects before deciding which patients to subject to a treatment protocol.

The review of the literature given above demonstrates that while plasma biomarkers have led to great progress in AD research, their application for identifying CAA has been limited but constitutes a promising arena. There is a need to study the existing long list of biomarkers and identify novel biomarkers that may help identify CAA. This approach should rely on samples well characterized via gold-standard quantitative assessments made using digital neuropathology along with antemortem data assessed using the Boston Criteria 2.0. The biomarker candidates identified need to be validated in a second cohort for which there are only antemortem imaging data assessed using the Boston criteria 2.0.

## 6. Limitations and Suggestions for Future Studies

Autopsy-based CAA identification is a gold standard, but it is not practical. The modified Boston criteria are helpful only in the presence of bleeding. To date, little research has been conducted to identify blood-based biomarkers for CAA. Detecting and staging CAA even before its consequences can be detected via neuroimaging are critical to allow clinicians to judiciously select appropriate candidates for AATs, stratify monitoring, properly manage therapeutic regimens for those experiencing symptomatic ARIAs, and optimize treatments to achieve the best outcomes. In our opinion, p-tau 217 and Aβ40 may have the greatest potential as CAA biomarkers because of the strong associations with Aβ accumulation and tau pathology, whereas PIGF may have the least potential as a CAA biomarker because of its indirect involvement in AD pathophysiology. However, more studies are necessary to establish the roles of the potential promising biomarkers we have reviewed in this paper. Future studies can test potential plasma biomarkers in human beings and evaluate the predictive value of individual markers for CAA severity.

## 7. Conclusions

CAA is a major risk factor for ARIA development. Identifying biomarkers for CAA is necessary for the proper management of AD. Post-mortem diagnosis is a definite means of diagnosing CAA, but it is not practical. The MRI-based Boston criteria are useful for diagnosing CAA, but they are only helpful after a hemorrhage has occurred. The current research on CAA biomarkers is centered on Aβ in CSF. The use of plasma-based biomarkers is a convenient and non-invasive strategy for detecting and staging CAA in its “preclinical” stage before its consequences are detected via neuroimaging. We propose further exploring the list of existing biomarkers and identifying novel biomarkers for the better prediction and early detection of CAA to minimize the risk of ARIAs in patients receiving AAT for the treatment and prevention of AD, an urgent global public health issue.

## Figures and Tables

**Table 1:** Progressive stages of Alzheimer’s Disease [1].

Stage 1	Stage 2	Stage 3	Stage 4	Stage 5
Preclinical AD	Mild cognitive impairment due to AD	Dementia due to AD: Mild	Dementia due to AD: Moderate	Dementia due to AD: Severe
No clinical symptoms but possible biological changes in the brain (e.g., abnormally increased accumulation of amyloid β and tau)	Subtle symptoms (e.g., problems with memory, language, and thinking) that may not interfere with daily activities	Symptoms interfering with some daily activities	Symptoms interfering with many daily activities	Symptoms interfering with most daily activities

**Table 2:** Results of CSF Aβ42, Aβ40, and Aβ40/42.

	Aβ42 (pg/mL) ^a^	Aβ40 (pg/mL) ^a^	Aβ40/42 ^a^
CAA	355 ± 146 (n = 17)	2912 ± 977 (n = 17)	9.43 ± 4.95 (n = 17)
AD	433 ± 135 (n = 72) ^d^	3713 ± 1081 (n = 72) ^c^	8.95 ± 2.57 (n = 72) ^b^
Controls	838 ± 253 (n = 58) ^e^	4003 ± 1185 (n = 48) ^c^	4.91 ± 1.13 (n = 48) ^e^

^a^ Mean ± Standard deviation, Statistical difference shown for CAA vs AD, CAA vs controls, ^b^ Not significanlty different, ^c^ *p* < 0.01, ^d^ *p* < 0.05, ^e^ *p* < 0.01.

**Table 3:** Summary of the literature on potential biomarkers.

Authors	Study Objective	Methods and Type of Study	Results
Banerjee G et al. (2020) [34]	To compare amyloid markers (Aβ_38_, Aβ_40_, Aβ_42_, sAβPP *α*, and sAβPPβ) and other markers in neurodegenerative disease (t-tau, p-tau, NFL, sTREM2, and neurogranin) in the CSF of patients with AD and CAA and control subjects (CSs), comprising 10 patients with CAA and 5 CS participants from the Biomarkers and Outcomes in Cerebral Amyloid Angiopathy study, making use of 20 samples from patients with AD and 5 samples from age-matched CS participants from the Specialist Cognitive Disorders Service at the National Hospital of Neurology and Neurosurgery: University College London Hospitals (UCLH) NHS Trust, London, UK	Exploratory hypothesis-generating study. Aβ_38_, Aβ_40_, and Aβ_42_ were measured using a Meso Scale Discovery V-PLEX Aβ peptide panel 1(6E10) kit. sAβPP*α* and sAβPPβ measured using a Meso-Scale Discovery sAβPP*α*/sAβPPβ Kit. Tau markers measured using *a* sandwich ELISA	The results of unadjusted analyses showed lower levels of all amyloid components measured (Aβ_38_, Aβ_40_, Aβ_42_, sAβPP *α*, and sAβPPβ) and higher NFL levels in participants with CAA. There were no differences in CSF t-tau, p-tau, sTREM2, or neurogranin profiles.
Rasing I et al. (2024) [68]	To investigate whether NFL and GFAP levels in serum and CSF were abnormal in CAA conditions in 187 participants (28 presymptomatic D-CAA mutation carriers, 29 symptomatic D-CAA carriers, 59 individuals with sporadic CAA, and 33 controls)	Cross-sectional study. NFL and GFAP levels were measured using Simoa™ and Simoa™ GFAP Discovery Kit.	Increased CSF GFAP levels were found in the early presymptomatic stage of CAA. Increased serum and CSF NFL and GFAP levels were found in either sporadic or more severe hereditary symptomatic stages of CAA.
Taylor X et al. (2020) [102]	To examine the association between TREM2 and early-stage CAA in a mouse model	Cross-sectional. Outcomes were measured based on mouse (9-month-old Tg-FDD and wild-type C57/BL6 mice) brain cells.	Decreased levels of TREM2 were found in early-stage CAA
Zhong R et al. (2024) [97]	To determine the impact of TREM2 deficiency on CAA and parenchymal Aβdeposition in the brain using Tg-SwDI mice	Cross-sectional. Outcomes were measured via immunohistochemical, immunofluorescent, and histochemical staining of mouse brain tissue.	There was a robust increase in amyloid load in the cortex, hippocampus, and thalamus following the loss of TREM 2. There was an association between loss of TREM2 and markedly reduced CAA levels in thalamus.
Moursel LG et al. (2019) [103]	To examine the association between osteoponin and vascular calcification (severe form of CAA) in 8 people with a hereditary form of CAA	Cross-sectional. Outcome was measured via immunohistochemistry of huma brain tissues	There was a correlation between the vascular accumulation of collagen 1 and osteoponin and CAA severity
